# Stabilizing active N species on support for enhancing ammonia synthesis

**DOI:** 10.1093/nsr/nwag097

**Published:** 2026-02-12

**Authors:** Lizhuo Wang, Ang Li, Yuhang Liang, Jia Ding, Hongwei Liu, Wei Li, Rongkun Zheng, Xiaodong Han, Xiaozhou Liao, Jun Huang

**Affiliations:** Laboratory for Catalysis Engineering, School of Chemical and Biomolecular Engineering, Sydney Nano Institute, The University of Sydney, Sydney 2006, Australia; Beijing Key Laboratory of Microstructure and Property of Advanced Materials, Beijing University of Technology, Beijing 100124, China; Laboratory for Catalysis Engineering, School of Chemical and Biomolecular Engineering, Sydney Nano Institute, The University of Sydney, Sydney 2006, Australia; School of Physics, The University of Sydney, Sydney 2006, Australia; Laboratory for Catalysis Engineering, School of Chemical and Biomolecular Engineering, Sydney Nano Institute, The University of Sydney, Sydney 2006, Australia; Australian Centre for Microscopy & Microanalysis, The University of Sydney, Sydney 2006, Australia; Beijing Key Laboratory of Microstructure and Property of Advanced Materials, Beijing University of Technology, Beijing 100124, China; School of Physics, The University of Sydney, Sydney 2006, Australia; Australian Centre for Microscopy & Microanalysis, The University of Sydney, Sydney 2006, Australia; Beijing Key Laboratory of Microstructure and Property of Advanced Materials, Beijing University of Technology, Beijing 100124, China; Department of Materials Science and Engineering, Southern University of Science and Technology, Shenzhen 518055, China; Australian Centre for Microscopy & Microanalysis, The University of Sydney, Sydney 2006, Australia; School of Aerospace, Mechanical and Mechatronic Engineering, The University of Sydney, Sydney 2006, Australia; Laboratory for Catalysis Engineering, School of Chemical and Biomolecular Engineering, Sydney Nano Institute, The University of Sydney, Sydney 2006, Australia

**Keywords:** ammonia synthesis, *in situ* environmental transmission electron microscopy, electron energy loss spectroscopy, micro-equilibrium, Ru/MgO

## Abstract

As an important chemical raw material, ammonia is of great significance in fertilizer production and hydrogen energy storage. Ammonia synthesis via the Haber–Bosch process has been under continuous development for over a century, with previous studies predominantly emphasizing the electron donation effect from the support. However, various phenomena hint at other support effects also contributing to this reaction, while the detailed mechanism is yet to be fully discovered. This research reveals an additional role of the supports and an alternative reaction pathway to boost the ammonia synthesis, based on *in situ* environmental transmission electron microscopy and electron energy loss spectroscopy (EELS) investigation of two selected model catalysts, Ru/MgO and Ru/Al_2_O_3_. The activation and dissociation of N_2_ and H_2_ molecules occurred on Ru particles to generate active N* and H* species. During the reaction, the *in situ*-generated oxygen defects on MgO act as an N* reservoir to form the Mg–N* interaction, as evidenced by both *in situ* EELS and diffuse reflectance infrared Fourier-transform spectroscopy. The density functional theory calculation confirmed that the dissociated N* over the Ru particle will automatically refill into the surface oxygen defects on MgO due to the lower refilling energy. Thus, the support not only extracts the active N* from the Ru surface by nitrogen spillover, thereby suppressing N* recombination to N_2_, but also promptly frees Ru active sites for subsequent activation. This strongly enhances the reaction rate of N_2_ activation and dissociation, and further boosts the ammonia productivity.

## Teaser

*In situ* microscopy reveals MgO acts as a ‘nitrogen reserve’ in Ru-based catalysts, temporarily storing nitrogen atoms, keeping ruthenium active and boosting ammonia production compared with Al_2_O_3_.

## INTRODUCTION

As an essential subject for the modern chemical industry, active nitrogen contained in ammonia is the cornerstone for many industrial products including fertilizer, explosives and polymers etc. In the past century, agricultural productivity increased more than 2-fold [1]. This tremendous achievement is driven by the development of the industrial ammonia synthesis process. Apart from producing fertilizer, ammonia is also considered as a promising hydrogen storage medium for its carbon-free and non-greenhouse-gas properties [2]. The hydrogen generated from renewable energy can react with dinitrogen and be stored in the ammonia. However, although there is more than 78 v% dinitrogen in the air, converting nitrogen from air to ammonia is not an easy process. The stable triple bond in the dinitrogen molecule hinders its utilization and this problem was not solved until the beginning of 20th century [3]. It was in 1908 that Haber developed the first economic-feasible protocol, the Haber–Bosch process, to convert the inert dinitrogen into ammonia directly [3]. An iron-based catalyst was applied in this process, as well as high pressure and temperature. Until now, the industrial ammonia synthesis method has still been based on the concept of the Haber–Bosch process, although some modification has been made [4].

The basic reaction of the Haber–Bosch process is shown below:


(1) \begin{eqnarray*}
{{\mathrm{N}}}_2 + 3{{\mathrm{H}}}_2 = 2{\mathrm{N}}{{\mathrm{H}}}_3 \quad {\mathrm{ \Delta H}} = - 46.1\ {\mathrm{kJ}}/{\mathrm{mol}}.
\end{eqnarray*}

The high pressure and temperature required by the reaction induce the process to be energy intensive [5]. Finding catalysts capable of driving the artificial ammonia synthesis more effectively under milder conditions is therefore of great significance. Some substantial research has been conducted by different groups over recent decades and some promising results have been produced. For example, Nørskov *et al*., using a density function theory (DFT) calculation, predicted that using an MoFe_6_S_9_ catalyst would enable good ammonia synthesis from dinitrogen [6]. Inoue *et al*. applied the flat Ru nanoparticle supported on a Ca(NH_2_)_2_ base in the reaction to convert dinitrogen and dihydrogen to ammonia with excellent synthesis efficiency as well as stability [7]. By introducing B–P pairs over a carbon nanotube, Chen *et al*. effectively converted the dinitrogen to ammonia via an electrocatalysis process [8]. Peng *et al.* combined Ru and Co and the bimetal catalyst displayed dual atomically dispersed active centres. The electro-driven Haber–Bosch process showed the possibility of generating a surface *NH_3_ intermediate with good efficiency when the temperature was as low as 50°C [9]. Very recently, Liu *et al*. determined that the anisotropic surface state of a BaAlSi electride will enhance the uniformity of Ru loading and the electron transfer efficiency between the support and Ru. The Ru/BaAlSi catalyst exhibited an ammonia synthesis rate of 550 mmol g_Ru_^−1^ h^−1^ at 400°C and 0.1 MPa [10]. Jiang *et al*. developed single-atom Fe-implanted N-doped carbon catalysts, which displayed a 558 μmol g_cata_^−1^ h^−1^ at 300°C at ambient pressure, making it closer to the ammonia synthesis reaction under mild conditions [11].

Among all of this research, however, most of the work is focusing on fabricating better active sites, which helps the dissociation of nitrogen and hydrogen, further accelerating the reaction rates, as well as reducing the required temperature [12]. As for the effect of the supporting material, most research attributes the prompting effect of the supporting material to enriching the electronic density of active sites [12]. Regarding the other effect of the support, it is hinted at by the theoretical calculation that the vacancies over metal oxides would be active for nitrogen dissociation [13]. An experimental study demonstrated that by exposing different MgO facets, the performance of ammonia synthesis over Ru/MgO catalysts would be varied [14]. Furthermore, another study illustrated that N vacancies on the LaN support in Ni/LaN is the active site for N_2_ dissociation, so that significantly reduces the activation energy of ammonia synthesis over Ni/LaN [15]. Some latest research expected that formation energies of nitride and hydride over a metal oxide support [16], and the metal oxide O 2p energy level [17] are potential descriptors to identify the ammonia synthesis performance. It has also been discovered by Bai *et al*. [18] that metal–support interaction would alter the coverage and stability of *H on the Ru nanoparticle, thereby influencing the ammonia synthesis process. All of these findings suggest the possibility that another important effect may be induced by the support material. However, until now, there has been limited evidence to reveal how the support effects the ammonia synthesis reaction.

This project is aimed at exploring the influence of the supporting material in ammonia synthesis. Considering Ru/MgO as one of the most popular ammonia synthesis catalysts [19–21], this research focuses on the discovery of the working geometry and surface sites of MgO supports during the ammonia synthesis via the combination of microscopic and spectroscopic investigations. The development of electron microscopy (EM), especially the *in situ* technology, takes the material characterization to a new dimension. It provides the opportunity to let the researchers to ‘look at the reaction’ in a micro-view and reveal the structural change of the material during the reaction. Additionally, analytical EM (AEM), for example electron energy loss spectroscopy (EELS), evidences the working chemical environmental and surface active centres for the catalysts. With the help of *in situ* EM and *in situ* AEM, some excellent work has been done on catalytic-related processes such as CO oxidation [22], metal oxide hydrolysation [23] and the metal sintering process [24] etc.

In this project, we applied *in situ* environmental transmission EM (ETEM) and EELS to analyse Ru/MgO as a model catalyst to investigate the MgO support effect on the ammonia synthesis reaction. The inert Al_2_O_3_ support was selected as a comparison. To highlight the support’s effect and avoid the influence of other species, no promoter was added to the system. *In situ* diffuse reflectance infra-red Fourier transform spectroscopy (DRIFTS) is further applied to verify our discovery.

## RESULTS

### Catalyst characterization and its performance

**Figure 1.**
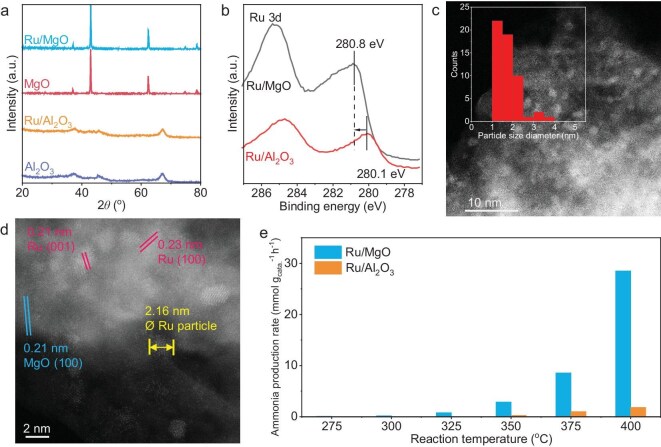
(a) Characterization and reaction performance of catalysts. XRD patterns for Ru/MgO, MgO, Ru/Al_2_O_3_ and Al_2_O_3_. (b) Ru 3d XPS spectra for Ru/MgO and Ru/Al_2_O_3_. (c) HAADF-STEM image of Ru/MgO and the particle size distribution. (d) High-resolution HAADF-STEM image of Ru/MgO. (e) Reaction performance of Ru/MgO and Ru/Al_2_O_3_ in ammonia synthesis reaction.

The X-ray diffraction patterns displayed in Fig. 1a indicate that both MgO and Ru/MgO have peaks located at 37.0°, 43.0°, 62.4° and 74.8°, as well as at 78.7°. These peaks correspond to the intrinsic MgO [PDF# 45-0946, *Fm-3m (225)*]. The characteristic diffraction patterns of room temperature stable hexagonal Ru (PDF# 06-0663) located at 38.4°, 42.1°, 44.0°, 58.3° etc. are not observed. A similar phenomenon is also observed over Al_2_O_3_ and Ru/Al_2_O_3_, where only the diffraction pattern of γ-Al_2_O_3_ [25,26] can be observed while that of Ru is not found. This result gives clues again that the size of the Ru particle is extremely small and outside the detection limit of X-ray diffraction (XRD) [27]. The X-ray photoelectron spectroscopy (XPS) results (Fig. 1b) provide the evidence that Ru is successfully loaded on both Ru/MgO and Ru/Al_2_O_3_ as the Ru 3d signal is detected in both samples. The first Ru 3d peak located at 280.1 eV for Ru/Al_2_O_3_ and the one for Ru/MgO displayed a negligible difference, locating at 280.3 eV. These results suggest that Ru nanoparticles on the MgO and Al_2_O_3_ surface are in the form of metallic Ru (Ru^0^) with similar electron status [28], which further hints that the electron donation effect between metal oxide and Ru nanoparticles may not be the key for altering ammonia synthesis activity in this system [29].

The Ru particles are also observed over Ru/MgO and Ru/Al_2_O_3_ via high-angle annular dark field- scanning transmission electron microscopy (HAADF-STEM) images (Fig. 1c and d, Fig. S1). As displayed in Fig. 1c and d, the bright Ru nanoparticles can be found over the bulk MgO. According to the imaging principles of HAADF-STEM, elements with higher atomic number (Z), such as Ru, will be brighter. On the contrary, the light elements of MgO will be darker in HAADF images [30]. The Ru nanoparticle displayed a hexagonal lattice, which fits the space group P63/mmc(194) of Ru and lattice parameters a = 0.269 nm and c = 0.427 nm. We measured the particle size of the Ru, and the distribution determines that most of these particles are smaller than 3 nm, which is smaller than the detecting limit of XRD (ca. 2.5–3 nm) [27]. The lattice distance of the MgO is 0.21 nm, which corresponds to the (200) panel of MgO. The lattice distances measured between bright particles are 0.21 and 0.22 nm, which are attributed to the Ru (101) and Ru (002) panels.

The ammonia production rate is displayed in Fig. 1e. No ammonia is detected at 275°C on both Ru/MgO and Ru/Al_2_O_3_. A trace amount of ammonia can be detected over Ru/MgO at 300°C, while the same amount of ammonia cannot be detected over Ru/Al_2_O_3_ until the temperature reaches 350°C. On both Ru/MgO and Ru/Al_2_O_3_, the reactivity is increased, along with the temperature elevation. For Ru/MgO, the ammonia synthesis rate was 2.86 mmol g_Ru_^−1^ h^−1^ at 350°C and this number jumped almost 10-fold to 28.5 mmol g_Ru_^−1^ h^−1^ when the reaction temperature reached 400°C. As for Ru/Al_2_O_3_, the ammonia productivity was 0.254 mmol g_Ru_^−1^ h^−1^ at 350°C and gradually increased to 1.81 mmol g_Ru_ h^−1^ at 400°C. Based on the activity result, we further calculated the activation energies of ammonia synthesis over Ru/MgO and Ru/Al_2_O_3_ in this experiment and the plot is displayed in Fig. S2 [31]. The apparent activation energy (E_a_) of ammonia synthesis over Ru/MgO is 158.7 ± 3.5 kJ/mol and the one over Ru/Al_2_O_3_ is 176.5 ± 24.1 kJ/mol.

### *In situ* ETEM/EELS investigation of the reaction surface on Ru/Al_2_O_3_ during ammonia synthesis

**Figure 2.**
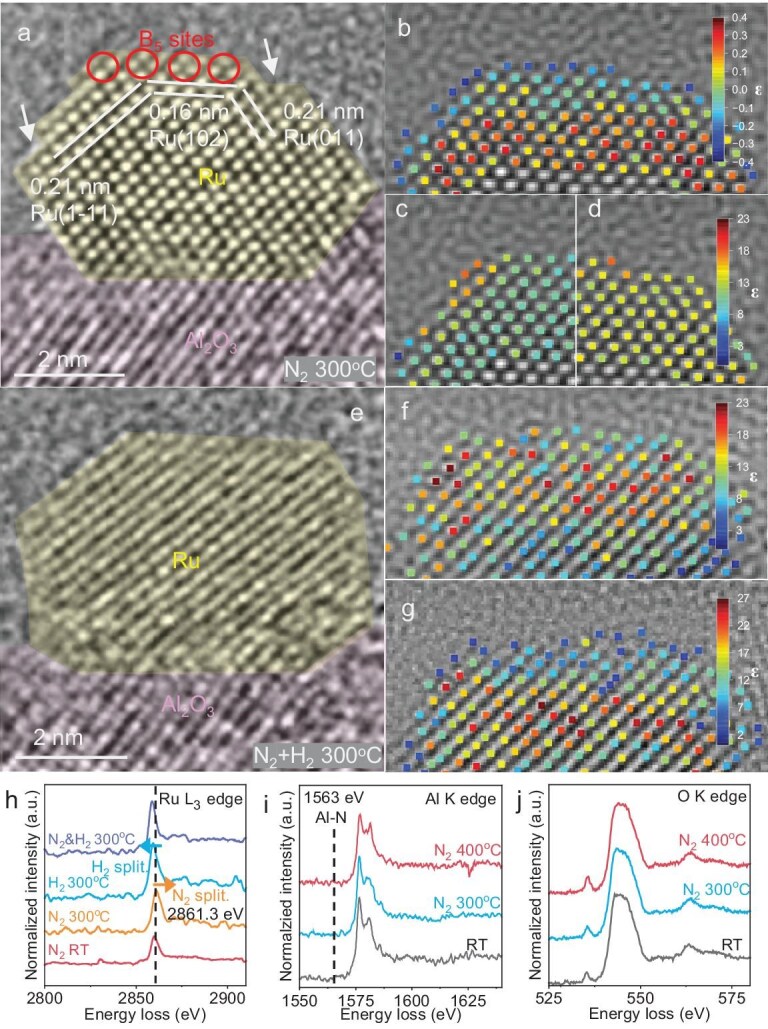
(a) *In situ* HRTEM and EELS analysis over Ru/Al_2_O_3_. The *in situ* HRTEM image of Ru/Al_2_O_3_ in an N_2_ atmosphere at 300°C for 0 s. The Ru nanoparticle is highlighted in yellow and Al_2_O_3_ is labelled in pink. (b) The estimated Ru atom column position and the number of Ru atoms in the corresponding column from the top of particle shown in (a) were identified through statistical parameter estimation. (c) The strain mapping of the Ru nanoparticle after exposure to N_2_ for 15 s in the *x*-direction. (d) The strain mapping of the Ru nanoparticle after exposure to N_2_ for 15 s in the *y*-direction. (e) *In situ* HRTEM image of Ru/Al_2_O_3_ in a H_2_ and N_2_ atmosphere at 300°C. The Ru nanoparticle is highlighted in yellow and Al_2_O_3_ is highlighted in pink. (f) The estimated Ru atom column position and the number of Ru atoms in the corresponding column of the Ru particle in (e). (g) *In situ* HRTEM image of Ru/Al_2_O_3_ exposed to H_2_ and N_2_ for a further 30 s and the estimated Ru atom column position and number of Ru atoms in the corresponding column. (h) *In situ* Ru K EELS spectra. (i) Al K EELS spectra. (j) O K EELS spectra acquired on Ru/Al_2_O_3_ under different conditions.

The *in situ* microscopy technique is applied to observe the geometry of the catalysts during the experiment. When the Ru/Al_2_O_3_ is placed in a hydrogen atmosphere under 300°C, the hydrogen dissociation is observed in the Ru *in situ* ETEM spectra, as shown in Fig. S4a and S4b in the Supplementary data [32–34]. The top-right edge of the Ru nanoparticle (as indicated by the lavender line) changed its structure from a stepped edge to a smooth edge. This structural change reflects the surface atomic movement of the Ru nanoparticle and may correspond to the lattice strain change of Ru [35,36] and crystal structural rearrangement [37] during the hydrogen activation. The Ru nanoparticle rearrangement was also observed at 300°C in an N_2_ atmosphere, as shown in Fig. 2a and d, and Fig. S5. The initial state of the Ru nanoparticle at 300°C and 0 s in N_2_ atmosphere shows the planes with lattice distances of 0.21, 0.16 and 0.21 nm (Fig. 2a). These planes are correspondingly assigned to (1-11), (102) and (011) planes of metallic Ru (ICSD# 40354). The stepped edges (highlighted by white arrows) and surface Ru B_5_ sites (indicated with red circles) are also observed on the Ru nanoparticle in Fig. 2a. The Ru columns in the nanoparticle are well aligned and arranged in an orderly fashion, as displayed in Fig. 2a and b. The Ru nanoparticle displays a well-defined morphology, and the estimated atom counting (Fig. 2b) of Ru atomic columns shows a clear gradient, with higher atomic occupancy in the particle core and progressively fewer Ru atoms toward the surface. After exposure to N_2_ at 300°C for 15 s, the strain mappings of the high-resolution TEM (HRTEM) image, shown in Fig. 2c and d, indicate the Ru atoms on stepped edges have a stronger displacement compared to Ru atoms in the bulk. We further measured the lattice distance of the Ru particle to verify this observation. The lattice spacing in Fig. S5 indicated by the light orange label measures 0.14 nm, aligning with the (102) plane of Ru. However, the top layer of the Ru (102) plane reveals a greater distance, specifically 0.18 nm, designated by the lavender colour. These surface geometry changes evidence the N_2_ dissociation on Ru since the published DFT calculation result suggests that N_2_ dissociative chemisorption over the Ru particle would lead to the rearrangement and lattice distance change on the particle surface [38]. Along with the proceeding of N_2_ dissociation, this surface dynamic change would even lead to the further shape rearrangement of the Ru particle, which is indicated in Fig. S6. For the Ru/Al_2_O_3_ reacted in N_2_ and H_2_, as shown in Fig. 2e–g, the rearrangement on the Ru outer layer persists. Exposure to a N_2_ and H_2_ mixture at 300°C incurred the disordered atom arrangement on the Ru nanoparticle surface, suggesting continuous N_2_ and H_2_ activation on Ru nanoparticles. The Ru nanoparticle in Fig. 2e was blurred as a slight rotation of the Ru nanoparticle occurred during the reaction, which may have originated from the interface reconstruction [39]. The estimated number of Ru atoms in individual atomic columns exhibits significant changes during the reaction (Fig. 2f and g), indicating that Ru atoms undergo redistribution within the Ru nanoparticle under reaction conditions. Meanwhile, the interface between Ru and Al_2_O_3_ got blurred when H_2_ and N_2_ were introduced simultaneously, further confirming that H_2_ would influence the interface structure of Ru/Al_2_O_3_ (Fig. S7).

The *in situ* Ru L_3_, Al K and O K EELS spectra shown in Fig. 2h–j depict the chemical status of the surface compositions being exposed to different conditions. Figure S8 indicates that N_2_ will not be activated at room temperature as there is no shift or shape change regarding the Ru L_3_ peak located at 2859.5 eV. After introducing N_2_ at 300°C the Ru L_3_ displayed a positive shift to 2861.3 eV, which suggested a lower electron density over the Ru particle [40,41]. Since nitrogen has strong electronegativity, the activation and dissociation of the dinitrogen molecule on the Ru particle surface could be the trigger of the Ru electron density decrease. On the contrary, if the sample was exposed to H_2_ at 300°C, the dissociated hydrogen pushed electrons towards metal particles, leading to a negative shift of the Ru metal peak. When N_2_ and H_2_ were added into the reactor together at 300°C, the negative shift can still be observed in the EELS Ru L_3_ spectrum, shown in Fig. 2h. It suggests that N_2_ splitting might be slower than H_2_ splitting on Ru, so that the Ru nanoparticle displayed the trend of being reduced when N_2_ and H_2_ were introduced together. In contrast to the peak movement of Ru L_3_, the Al K and O K spectra of Ru/Al_2_O_3_ didn’t exhibit peak position change. The Al–N peak located at 1563 eV [42] was not observed and the O–K peak remains a steady shape despite the conditions having changed.

### *In situ* ETEM/EELS investigation of reaction surface on Ru/MgO during ammonia synthesis

**Figure 3.**
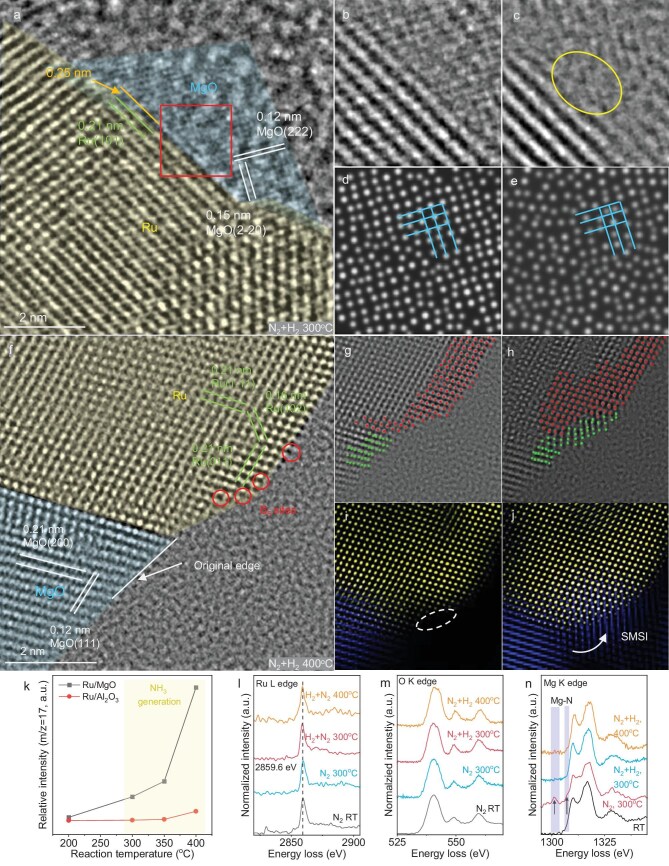
(a) *In situ* HRTEM image of Ru/MgO heated in N_2_ and H_2_ at 300°C for 0 s. Ru is highlighted in yellow and MgO is highlighted in blue. The red square indicates the edge region between Ru and MgO, which is further analysed in (b–e). (b) TEM image of the interface between Ru and MgO indicated in (a). (c) same region to (b) after exposure to N_2_ and H_2_ at 300°C for 120 s. (d) Contrast-intensified FFT-filtered image of (b). (e) Contrast-intensified FFT-filtered image of (c). (f) *In situ* HRTEM images of Ru/MgO heated in N_2_ and H_2_ at 400°C for 0 s. Ru is highlighted in yellow and MgO is highlighted in blue. (g) *In situ* TEM images when Ru/MgO is heated in N_2_ and H_2_ at 400°C for 0 s. (h) *In situ* TEM image when Ru/MgO is heated in N_2_ and H_2_ at 400°C for 50 s. (i) Fake-coloured FFT-filtered image of (g). (j) Fake-coloured FFT-filtered image of (h); blue represents MgO and yellow represents Ru. (k) MS intensity of *m*/*z* = 17 acquired from ETEM discharge gas when observing Ru/MgO and Ru/Al_2_O_3_. (l) Ru L EELS results. (m) O K EELS results. (n) Mg K EELS results of Ru/MgO acquired under different conditions.

As shown in Fig. S9 and Fig. 3a, the outer layer of the Ru (101) panel expanded from 0.21 nm to 0.25 nm in N_2_ or N_2_ and H_2_ at 300°C. The MgO in the Ru/MgO interface displayed a clear lattice panel with distance 0.12 and 0.15 nm (Fig. 3a and b), corresponding to the (222) and (2-20) lattice panels, respectively. After being exposed for 120 s in N_2_ and H_2_ at 300°C (Fig. 3c), MgO close to Ru was blurred, indicating the amorphization of this site (indicated by the yellow circle). The fast Fourier transform (FFT) filter was applied to these edge sites, as shown in Fig. 3b and c, for removing the influence from the background and Ru. The peak-finding function in StatSTEM was further implemented to intensify the contrast of the FFT-filtered images and the results are shown in Fig. 3d and e. It is shown that most of the dots in Fig. 3d are periodically distributed and aligned well with the lattice, indicative of a crystalline structure. In contrast, Fig. 3e shows a random distribution pattern, suggesting that the MgO at the Ru/MgO interface undergoes a transition from a crystalline to an amorphous structure during the reaction. Also, the FFT images of the interface between Ru and MgO in Fig. 3b and c are shown in Fig. S10. The diminishing of the MgO FFT pattern double confirms the amorphous site generation on MgO at the interface. The generation of the amorphous region and the rough edge suggests that the oxygen depletion defects (i.e. oxygen vacancies, O_v_) were accumulated on the surface of MgO under the effect of H_2_ [43,44], and the amorphous site is also observed on Ru/MgO treated with H_2_ (Fig. S11): the H* chemical adsorbed on the MgO surface generates superficial –OH groups and left oxygen vacancies after removing these –OH groups by forming water [45,46]. These results are consistent with the electron paramagnetic resonance (EPR) and hydrogen temperature-programmed reduction (H_2_-TPR) results shown in Figs S12 and S13. The MgO geometry kept changing at 400°C with H_2_ and N_2_ (Fig. 3f–j). In Fig. 3f, three planes on the Ru nanoparticle with lattice distances of 0.21, 0.16 and 0.21 nm were identified and corresponded to (1-11), (102) and (011) planes of Ru (ICSD# 40354). This determines that the Ru nanoparticle on MgO has the same exposure surface compared to Ru on Al_2_O_3_ and, similarly, a considerable number of the Ru surface B_5_ sites (indicated by red circles) were also observed on Ru/MgO, which may be a benefit for N_2_ activation. The HRTEM images (Fig. 3g and h), as well as the corresponding fake-colour FFT filter images (Fig. 3i and j), illustrate the reconstruction of the MgO edge at the Ru/MgO interface. MgO domains are observed to be redistributed towards the Ru nanoparticle and partially encapsulate the Ru particle during the ammonia synthesis reaction, which contributes to the strong metal-support interaction (SMSI). This movement suggested that the MgO surface is reconstructed during the reaction by generation of defects and refilling. The FFT image of this Ru/MgO interface being exposed in N_2_ and H_2_ at elevated temperature, shown in Fig. S14, confirmed the co-existence and changing of Ru and MgO. The mass spectrometry (MS) results of gas discharged from ETEM (Fig. 3k) shows an increased ammonia intensity when the temperature increased to 300°C, suggesting the light-off temperature is around 300°C for this reaction in the sample. The MS spectra given in Fig. S15 confirm that ammonia was produced.

The *in situ* Ru L EELS spectra shown in Fig. 3l do not reveal an obvious shift when Ru/MgO is exposed to N_2_ at 300°C. This Ru L_3_ peak is located at 2859.6 eV, whereas the one on Ru/Al_2_O_3_ shifted to 2861.3 eV when exposed to the same condition. It may be caused by the less-dissociated N on the Ru surface of Ru/MgO, compared to that on Ru/Al_2_O_3_, since the electronegative N impacts the Ru electron density. In *in situ* O K EELS spectra (Fig. 3m), the Ru/MgO displayed an O K peak at 540.3 eV at room temperature in N_2_. No O K EELS result change was identified when the reaction condition changed. It is important to notice that the Mg–N peaks located around 1302 and 1307 eV were observed in Mg K EELS spectra (Fig. 3n) of Ru/MgO at 300°C in N_2_, [47] determining a newly generated Mg–N interaction on MgO. It indicates that the N* activated on the Ru surface was transported to oxygen vacancies on the MgO surface by nitrogen spillover. This transportation on Ru/MgO resulted in the N* concentration reduction on the Ru particle. Meanwhile, the N* in MgO oxygen vacancies generates Mg–N bonding on the MgO surface, as shown in Mg K EELS spectra. Further exposing the sample to H_2_ at 300°C or 400°C, the Mg–N signal disappeared in the Mg K spectrum due to consumption with H_2_ to form ammonia. This N* transportation and reaction on MgO also explains the Ru/MgO interfacial dynamic on TEM images; this N* transportation and reaction continuously generates and consumes Mg–N on the MgO surface, resulting in a circle of generating and refilling of MgO surface defects. This also provides a possible mechanism of the aforementioned SMSI formation. Conventionally, the SMSI is predominantly observed on reducible metal oxides such as CeO_2_ and TiO_2_, whereas non-reductive metal oxides, such as MgO, are generally considered incapable of SMSI. The observed SMSI on Ru/MgO in this work indicates dynamic reconstruction of the MgO surface during the reaction, involving the generation and subsequent refilling of surface defects. This finding implies that the intrinsic reducibility alone might not be the decisive factor that governs SMSI. Instead, the ability of the support to undergo a cyclic process of vacancy generation and annihilation may be the key prerequisite for SMSI formation. Because reducible metal oxides more readily facilitate such defect dynamics, SMSI has traditionally been associated exclusively with this class of materials.

### Improvement of ammonia productivity

There are two primary mechanisms for ammonia synthesis: the dissociative mechanism; and the associative mechanism. The specific mechanism followed is highly dependent on the active site structure. The associative mechanism is favoured on Ru single atoms and inert flat surfaces that lack Ru B_5_ sites [12,48]. Conversely, for Ru-based catalysts with B_5_ sites, the dissociative mechanism is preferred, as the B_5_ sites significantly reduce the activation energy for N_2_ dissociation [49]. In this project, Ru nanoparticles with an average size of ca. 2 nm are optimal for the formation of B_5_ sites [50], and the TEM results confirmed that both samples contain considerable numbers of B_5_ sites on the Ru nanoparticle surface. Consequently, it is theoretically more likely that the dissociative mechanism dominates the reaction. In this pathway, dinitrogen dissociates over the Ru B_5_ sites, with this step being the rate-determining step [50]. This aligns with the observed phenomenon that Mg–N is consumed when H_2_ participates in the reaction, as the generated N* are immediately consumed. The N_2_ dissociation process over Ru is reversible [51]. As given in Equation 4, the dissociated N* may combine with each other to regenerated N_2_ over the Ru particle and be released into gas phase. This recombination process hinders the overall efficiency of dinitrogen dissociation and further influences the outcome of ammonia synthesis. From this prospect, the transportation of activated N* from Ru particle to MgO surface reduces the N* concentration on Ru surface, which drives the equation (4) moving toward right side. Ru on Ru/MgO is only responsible for H_2_ and N_2_ dissociation, while the formation of ammonia occurred on MgO. On the contrary, as for Ru/Al_2_O_3_, much more activated N* on Ru particle provides higher opportunity for N* recombination, which limited the ammonia generation.


(4) \begin{eqnarray*}
{{\mathrm{N}}}_2 \leftrightarrow {{\mathrm{N}}}_{2,{\mathrm{ads}}} \leftrightarrow 2{{\mathrm{N}}}^*.
\end{eqnarray*}

The nitrogen temperature-programmed desorption (N_2_-TPD) double confirms the balance movement over the catalyst. The results in Fig. S16 show that Ru/MgO has a better N_2_ chemisorption capacity. A main desorption peak at 316°C can be seen on the Ru/MgO catalyst, while obviously weaker peaks are observed on Ru/Al_2_O_3_ with the same Ru loading. This result suggests that Ru/MgO has a larger capacity for N_2_ chemisorption than Ru/Al_2_O_3_. For pure MgO and Al_2_O_3_, they show a broad desorption peak at 200°C–500°C with similar peak intensity, indicating that the MgO and Al_2_O_3_ supports have limited N_2_ adsorption capacity.

The *in situ* DRIFTS spectra exhibit further evidence for our assumption and reveal the mechanism of the overall reaction. As displayed in Fig. S17, after adsorption with ammonia on Ru/MgO as a reference, the surface Mg–NH_2_ and Mg–NH peaks can be observed at 3294 and 3220 cm^−1^ [52]. These two peaks are also observed over Ru/MgO if N_2_ and H_2_ are introduced together in the chamber at 300°C, indicating that ammonia was generated on the surface of MgO. This is consistent with the EPR results (Fig. S12) that no g = 2.001 peak was observed on the spent Ru/MgO. Furthermore, the broad peak at 3520 cm^−1^ is observed on Ru/MgO in the same conditions [53], which is attributed to the stretching vibration of surface –OH species. This surface –OH group may originate from the hydrogen spillover on the MgO surface. Furthermore, a weak peak located at 1993 cm^−1^ was attributed to the N_2_ adsorbed on the Ru surface [54]. No peak was observed between 2300 and 1900 cm^−1^, indicating that no N_2_ was directly adsorbed on the MgO [55]. The fresh and spent Ru/MgO catalysts were further analysed by STEM energy dispersive X-ray spectroscopy (STEM-EDS) mapping. As shown in Fig. S18, a pronounced nitrogen signal is observed on the MgO in the spent catalyst, whereas no discernible nitrogen distribution is detected in the fresh Ru/MgO sample. Also, the STEM-EDS line-scan results determine that the Ru and N signals are spatially separated on the spent Ru/MgO, providing further support for the proposed mechanism.

**Figure 4.**
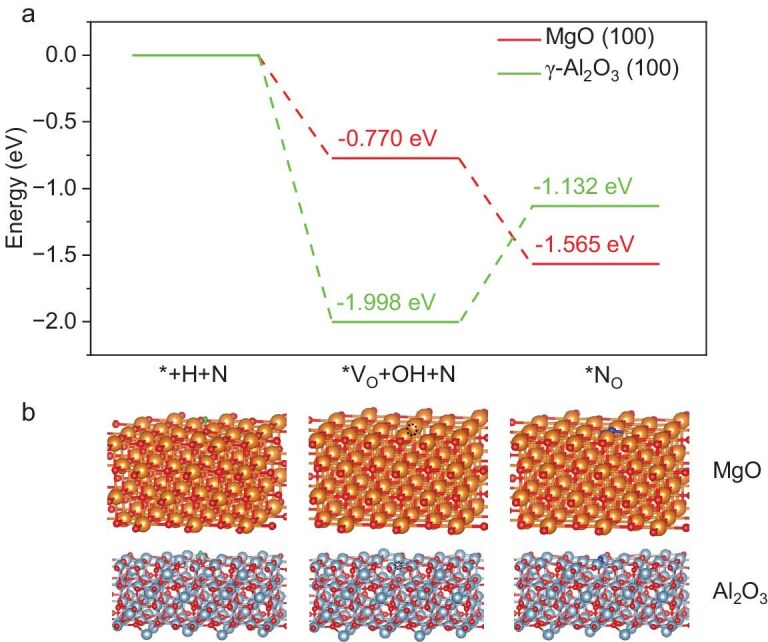
(a) DFT calculation results. Formation energy of generation of surface oxygen vacancies and refilling of the oxygen vacancies with activated N* on MgO and Al_2_O_3_ surfaces. (b) The illustration of surface generation of oxygen vacancies and refilling with activated N* on MgO and Al_2_O_3_ surfaces.

**Scheme 1.**
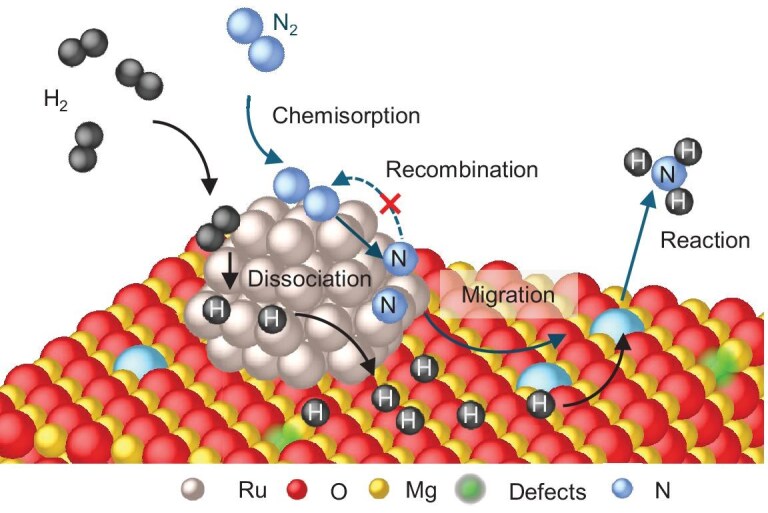
The alternative pathway via Mg–N* for ammonia synthesis on Ru/MgO.

To validate the observed activated N* on MgO defects, a DFT calculation was implemented. The DFT results (Fig. 4) indicate that the formation energies for the formation of oxygen vacancies on MgO and Al_2_O_3_ are −0.770 and −1.998 eV, respectively. Interestingly, the oxygen defects on Al_2_O_3_ and MgO show different behaviour against N* interaction. For MgO, the formation energy of refilling activated N* to oxygen defects was −1.565 eV, which is much smaller than −0.770 eV of the defect formation. Thus, the N* could automatically refill into the surface oxygen defects on MgO. At the same time, the H_2_ is dissociated over Ru nanoparticles and forming H* [56]. This H* could be further transported to the MgO surface adjacent to the Ru nanoparticle via hydrogen spillover [57,58]. Consequently, the activated N*-in-vacancy will react with spillover hydrogen on the MgO surface and turn to N–H, finally converting to ammonia. On the contrary, as for Al_2_O_3_, the interaction energy of N* with the oxygen defect is –1.132 eV, which is much higher than the formation energy of generating oxygen vacancies on Al_2_O_3_. Therefore, refilling N* into oxygen vacancies on Al_2_O_3_ is unfavourable, which hinders the movement of N* to the surface oxygen vacancies on Al_2_O_3_. Accordingly, the ammonia synthesis route over Ru/MgO is illustrated in Scheme 1 and the different pathway over Ru/Al_2_O_3_ is displayed in Scheme S1.

## CONCLUSIONS

This research discovered an additional role of the supports and an alternative reaction pathway for enhancing ammonia synthesis, revealed through *in situ* ETEM and EELS investigations of two model catalysts, Ru/MgO and Ru/Al_2_O_3_, under identical reaction conditions. The observed surface dynamics and chemical environmental change of Ru confirm that the activation of N_2_ and H_2_ molecules occurs on Ru particles. The spillover of H from Ru to the MgO support can remove the lattice oxygen on MgO and result in the rearrangement of the MgO edge to an amorphous structure, which generates a large number of oxygen defects on surface. The *in situ*-generated oxygen defects on MgO would be replenished by activated N* spilled over from the Ru nanoparticle surface, and this cyclic process of vacancy formation and refilling induces SMSI on the non-reducible MgO support. As confirmed by DFT calculation, the dissociated N* over the Ru particle will automatically refill into the surface oxygen defects on MgO due to the lower refilling energy of −1.565 eV. Thus, the MgO support not only extracts the active N* from the Ru surface, restraining the recombination of N* to N_2_ via changing the reaction equilibrium of dinitrogen dissociation, but also quickly releases the Ru active sites for the activation of the next coming N_2_ molecules. Thus, the reaction rate of N_2_ activation and dissociation should be strongly enhanced. The active N* stabilized by the oxygen defect of MgO will further react with the active H* spilling over from the Ru surface for boosting ammonia productivity, while for the supports, which are poor in stabilization of the active N* due to the higher energy, the N* generation rate would be strongly limited by the recombination of N* on Ru sites and the reduced number of available Ru sites for the N_2_ activation and dissociation. It results in Ru/MgO having overwhelmingly better performance in ammonia synthesis compared to Ru/Al_2_O_3_ in this research.

## METHODS

### Synthesis of catalyst

The catalyst synthesis is separated to two steps and the supporting MgO is home-made, based on the published method [59]. The Ru/MgO catalyst is synthesized via a typical impregnation method. The resultant powder was dried in a dehydrating oven and then calcinated in a muffle furnace at 550°C for 3 h. The theoretical Ru content is 5 w%. Before the ammonia synthesis experiment, the catalyst is reduced in H_2_ at 400°C for 1 h. For comparison, the γ-alumina support Ru is synthesized via the similar procedure and denoted by Ru/Al_2_O_3_.

### Catalytic performance test

The ammonia synthesis activity was measured on a stainless-steel fixed-bed reactor under a continuous flow of syngas. The ammonia production rate was measured by using a conductivity meter, and the exhaust gas was conducted to a diluted sulphuric acid solution (1 mM).

### Catalyst characterization

The XRD pattern was collected by a PANalytical X’pert PRO powder diffractometer. HAADF-STEM images were taken on an FEI Themis Z equipped with a probe and image spherical aberration correctors. XPS was carried out on an ESCALAB250Xi spectrometer (Thermo Scientific). H_2_-TPR and N_2_-TPD experiments were conducted on a Quantachrome ChemBET Pulsar. The EPR experiments were carried out using a Bruker EMXnano EPR spectrometer operated at 298.15 K.

The *in situ* TEM was conducted using spherical aberration-corrected ETEM (FEI) accompanied by a DENSsolutions^®^ Lightning sample holder. During the experiment, the dosage of the electron was limited below 700 e/Å^2^ to minimize the influence of the electron beam on the sample. Atom column position estimation and contrast peak finding of the images were conducted using StatSTEM software [60]. *In situ* TEM-EELS spectra were acquired together with acquisition of *in situ* ETEM images in a similar situation. The TEM-EELS was acquired at 27 000× magnification and the irradiation area was around 100 μm^2^. The EELS signal was captured from the entire illuminated field. A Gatan^®^ electromagnetic prism was applied to generate EELS spectra. For ETEM observation over Ru/Al_2_O_3_ and Ru/MgO, the mass spectra for the exhaust from ETEM were acquired using an online MS spectrometer. The *in situ* DRIFTS spectra were collected using a Thermo Fisher^®^ Nicolet iS50 FT-IR with a Harrick DRIFTS cell and heating unit.

### DFT calculation

The spin-polarized DFT calculations [61] were performed as implemented in the Vienna *ab initio* simulation package (VASP) [62]. The DFT-D3 scheme of Grimme was adopted for the van der Waals correction [63]. For the exchange and correlation functional, the Perdew–Burke–Ernzerhof (PBE) [64] form of the generalized gradient approximation (GGA) was used to describe the electron–ion interaction in the projector augmented-wave approach [65], and the spin–orbit coupling (SOC) was included. The plane wave cutoff was set at 500 eV. The bulk lattice constants were optimized with a Monkhorst–Pack sampling of 12 × 12 × 12 and 10 × 10 × 10 k-point grids for the MgO and γ-Al_2_O_3_ unit cells, respectively. Surface calculations were performed with a Monkhorst–Pack sampling of a 2 × 2 × 1k-point grid. All the geometries were fully relaxed until the forces on each atom were less than 0.01 eV/Å.

## Supplementary Material

nwag097_Supplemental_File
