# Supplementary material for: Stabilizing active N species on support for enhancing ammonia synthesis
Source: Natl Sci Rev. 2026 Feb 12;13(7):nwag097. doi: 10.1093/nsr/nwag097 (PMC13056710; doi:10.1093/nsr/nwag097)
Supplement: nwag097_Supplemental_File [file nwag097_supplemental_file.pdf]

Supporting Information

of

# Stabilizing Active N Species on Support for Enhancing Ammonia Synthesis

Lizhuo Wang[1], Ang Li[2,*], Yuhang Liang[1,3], Jia Ding[1], Hongwei Liu[4], Wei Li[2], Rongkun Zheng[3,4], Xiaodong Han[2,6,*], Xiaozhou Liao[4,5], Jun Huang[1,*]

[1] Laboratory for Catalysis Engineering, School of Chemical and Biomolecular Engineering, Sydney Nano Institute, The University of Sydney, NSW 2006, Australia

[2] Beijing Key Laboratory of Microstructure and Property of Advanced Materials, Faculty of Materials and Manufacturing, Beijing University of Technology, Beijing, 100124, China

[3] School of Physics, The University of Sydney, Sydney, NSW, 2006, Australia

[4]Australian Centre for Microscopy & Microanalysis, The University of Sydney, Sydney, NSW 2006, Australia

[5] School of Aerospace, Mechanical and Mechatronic Engineering, The University of Sydney, Sydney, NSW 2006, Australia

[6] Department of Materials Science and Engineering, Southern University of Science and Technology, Shenzhen, 518055, China

[*]corresponding authors: ang.li@bjut.edu.cn; xdhan@bjut.edu.cn; jun.huang@sydney.edu.au

## 1. Detailed methods

### Synthesis of catalyst

The catalyst synthesis is separated to two steps. The supporting MgO is home-made based on the published method [1]. Typically, proper amount of $Mg(NO_3)_2$ was dissolved into the pure water. The concentration of the solution is controlled to be 1.5 mol/L. Then, 1 mol/L sodium carbonate ($Na_2CO_3$) solution is added to the $Mg(NO_3)_2$ solution dropwise with continuous stirring until all of $Mg^{2+}$ ions are precipitated. Next, the 10 mol/L NaOH solution is added into the mixture and the pH value is adjusted to around 10. The resultant slurry is then aged in an 80°C over for 12 hours. The mixture is then filtrated and washed by deionized water for several times and then dried in a 120°C oven for another 10 hours. After drying, the solid is crushed into small pieces by mortar and calcinated in muffle furnace at 850°C for 5 hours in the air. The resultant white powder is home-made MgO support.

The Ru/MgO catalyst is synthesised via a typical impregnation method. For example, 0.102g $RuCl_3$ is dispersed in 25 ml deionized water in a beaker. Then 1.00 g homemade MgO is added into the beaker and the mixture is stirred for 1 hour before the water in the mixture is evaporated

on a heat plate. The resultant powder is then dried in a dehydrate oven and calcinated in muffle furnace at 550°C for 3 hours. The theoretical Ru content is 5w%. Before the ammonia synthesis experiment, the catalyst is reduced in $H_2$ at 400°C for an hour. The catalyst is denoted as Ru/MgO. For comparison, the γ-alumina support Ru is synthesised via the similar procedure and denoted as Ru/$Al_2O_3$.

**Catalytic performance test**

The ammonia synthesis activity was measured on a stainless-steel fix-bed reactor under a continuous-flow of syngas. Typically, 100 mg Ru/MgO were packed layer-up-layer into a stainless-steel tube reactor (i.d. 8 mm). The test was carried out at elevated temperature under the given pressure (1Mpa) and flow rate ($N_2$:$H_2$ = 15sccm:45sccm). The ammonia production rate was measured by using a conductivity meter (HANNA Instruments, HI98192). The exhaust gas was conducted to a diluted sulfuric acid solution (1 mM) and the change in proton conductivity with time was calculated.

**Catalyst characterisation**

The XRD pattern was collected over PANalytical Xpert Pro powder diffractometer (45 kV and 40 mA) using Cu $K_\alpha$ radiation (λ= 1.5405 Å). The scanning range for all of the sample was 5° to 80°. High angle annular dark filed- scanning transmission electron microscopy (HAADF-STEM) images were token on the FEI Themis Z equipped with probe and image spherical aberration correctors. The operation high tension was 300 kV. X-ray photoelectron spectroscopy (XPS) was carried out on an ESCALAB250Xi spectrometer (Thermo Scientific, U.K.) with a monochromate Al Kα X-ray source (E = 1486.68 eV). $H_2$-TPR and $N_2$-TPD experiments were conducted on Quantachorme ChemBet Pulsar. The EPR experiments were carried out in Bruker EMXnano EPR Spectrometer operated at 298.15K.

The *in situ* TEM was conducted over image spherical aberration corrected FEI Environmental-TEM (ETEM) companied with DENSESolution® Lightning sample holder. Nitrogen and hydrogen were the reactant gas and leaked into the ETEM. The molar ratio between nitrogen and hydrogen was 1:3 and the reaction pressure were set to around $1\times10^{-1}$ mbar. During the experiment, the dosage of the electron was limited below 700 e/Å$^2$ to minimize the influence of electron beam to the sample. Besides, the sample was irradiated under same beam for 60 mins without reactant gases input to guarantee the implemented electron beam will not induce the atom rearrangement on the sample. The ETEM images shown

on Figure S3 for samples before and after electron beam irradiation determine minimal structural change can be observed, which verified the beam induced structural change to the sample is negligible. Atom column position estimation and contrast peak finding of the images were conducted over StatSTEM software [2]. For the contrast-intensified FFT filtered image, the high intensity lattice which influences analysis was firstly removed by FFT filter. The gaussian fitting was then applied to the resultant image to find the high-contrast area. *In situ* TEM-EELS spectra are acquired together with *in situ* ETEM images' acquiring in similar situation. The TEM-EELS was acquired at 27,000X magnification and the irradiation area was around 100 $um^2$. EELS signal was captured from the entire illuminated field. Gatan® electromagnetic prism is applied to generate EELS spectra. When ETEM observation over $Ru/Al_2O_3$ and Ru/MgO, the mass spectra for the exhaust from ETEM are acquired by online MS spectrometer with *in situ* TEM under 'Faraday Airdemo Scan Stair' mode in the m/z range of 0-50.

The *in situ* DRIFTS spectra were collected on ThermoFisher® Nicolet iS50 FT-IR with DRIFTS cell and heating unit. Before the reaction, the catalyst will be heated in Ar at 500°C for 30 minutes to remove the adsorbed surface species. The cell was then cooled down to room temperature before 60 sccm diluted nitrogen and hydrogen (88% Ar balance, molar ratio $N_2$ : $H_2$ = 1:3). For the signal collection, an MCT-A detector refrigerated by liquid nitrogen was applied. The scan number as 128 times and the resolution of the spectrum was 4 $cm^{-1}$.

**DFT calculation**

The spin-polarized density functional theory (DFT) calculations [3] were performed as implemented in the Vienna ab initio simulation package (VASP) [4]. The DFT-D3 scheme of Grimme was adopted for the van der Waals correction [5]. For the exchange and correlation functional, the Perdew-Burke-Ernzerhof (PBE) [6] form of the generalized gradient approximation (GGA) was used to describe the electron-ion interaction in projector-augmented wave approach [7], and the spin-orbit coupling (SOC) was included. The plane wave cutoff was set at 500 eV. The bulk lattice constants were optimized with a Monkhorst–Pack sampling of 12×12×12 and 10×10×10 k-point grid for the MgO and $\gamma$-$Al_2O_3$unit cell, respectively. The MgO(100) and $\gamma$-$Al_2O_3$ (100) surface models were constructed by 3×3 slabs of 4 layers separated by 15 Å of vacuum in periodic cells. Surface calculations were performed with a Monkhorst–Pack sampling of 2×2×1k-point grid. All the geometries were fully relaxed until the forces on each atom are less than 0.01 eV/Å.

## 2. Activation energy calculation

The activation energy of ammonia synthesis was calculated based on Arrhenius equation (equation (S1))

$$k = Ae^{-\frac{Ea}{RT}} \tag{S1}$$

Where k is the rate constant, A is the pre-exponential factor, Ea is the activation energy (J/mol), T is the reaction temperature (K), and R is the universal gas constant, which is 8.314J/(K·mol).

If we were taking the natural logarithm for both sides of the equation, then the equation can be expressed as the form below:

$$lnk = lnA - \frac{Ea}{R}\frac{1}{T} \tag{S2}$$

If we plot the lnk as the function of (1/T), then we will obtain a line and the slope of which is equals to –(Ea/R). The activation energy can be calculated.

## 3. Supporting figures

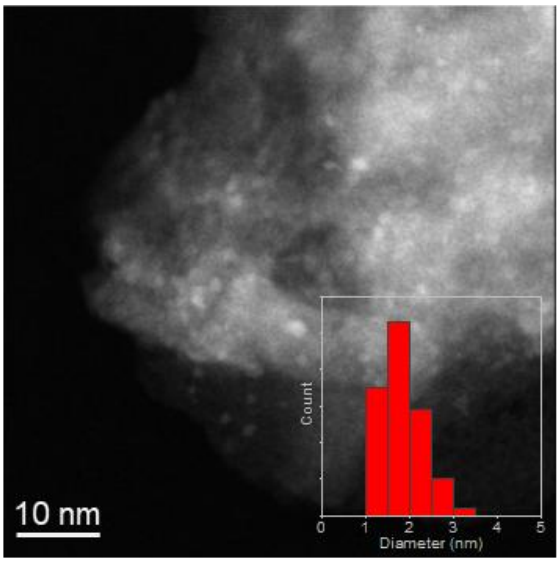


Figure S1 The HAADF-STEM image of Ru/$Al_2O_3$

According to the XRD and HAADF-STEM images given on Figure 1 and Figure S1, it is noticed that Ru particle size on Ru/MgO and Ru/$Al_2O_3$ are similar. The particle size distribution from HAADF-STEM images indicated the median Ru particle size on $Al_2O_3$ (1.8 nm) is similar to the one on MgO (1.6 nm). Considering the metallic Ru atom size is 134 pm [8], the 1.8 nm equivalent to around 13 Ru atoms' length while the 1.6 nm equivalent to around 12 Ru atoms' length. Assuming the Ru nanoparticles are in the form of sphere, the dispersion of Ru atoms for 1.6 nm nanoparticle is 0.81 and for 1.8 nm nanoparticle is 0.72[9]. Thus, the theoretical active sites number over Ru/MgO to Ru/$Al_2O_3$ is similar. From the ammonia synthesis activity given on Figure 1, the conversion rate over Ru/MgO is 10 times compared to Ru/$Al_2O_3$. This significant different activity over Ru/MgO and Ru/$Al_2O_3$ is difficult to be fully attributed to the active sites difference.

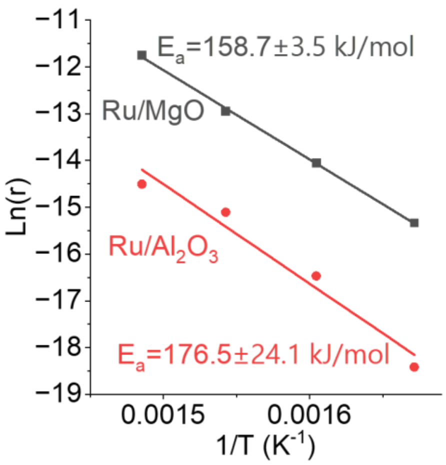


Figure S2 the plot for nature longitude of apparent reaction rate as a function of 1/T. Experiment pressure: 10 bars.

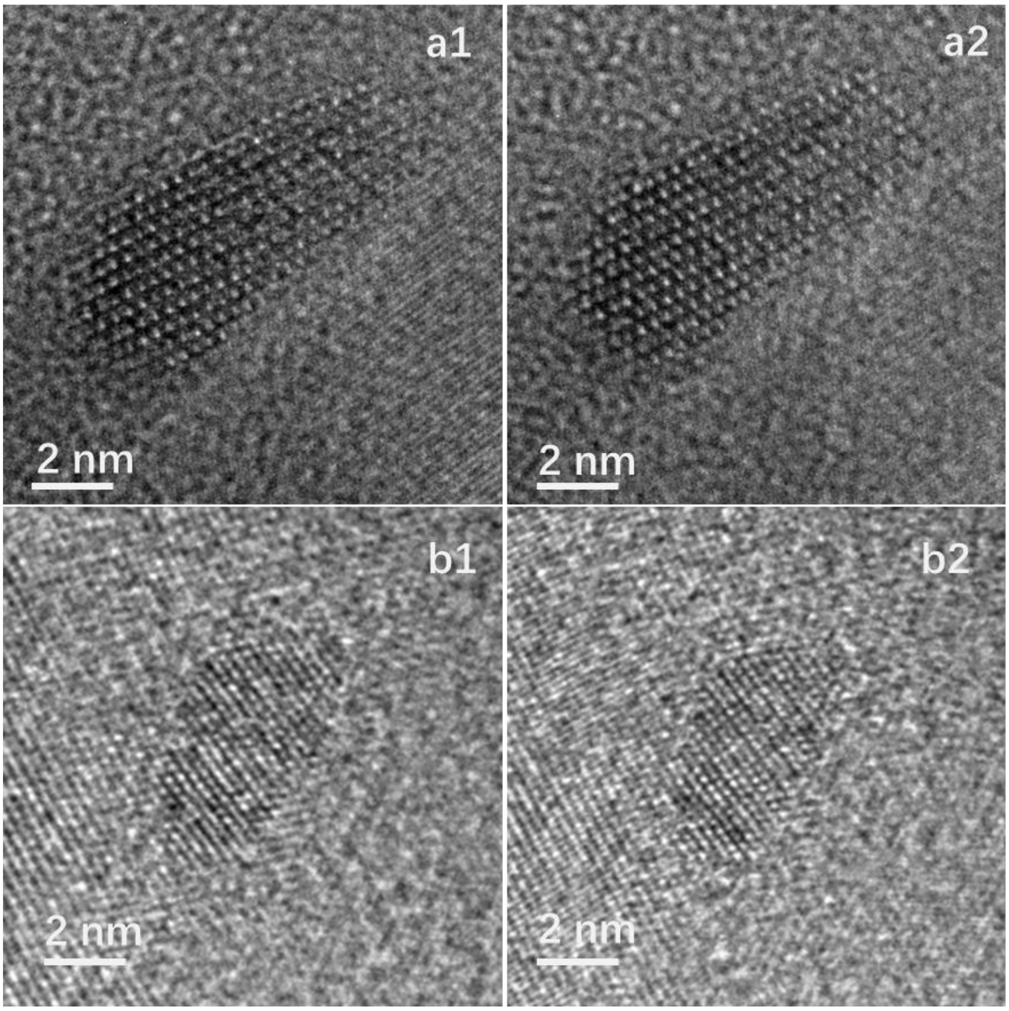


Figure S3 the *in situ* ETEM images of Ru/$Al_2O_3$ before (a1) and after electron beam irradiation (a2) in vacuum at room temperature; the *in situ* ETEM images of Ru/MgO before (b1) and after (b2) electron beam irradiation in vacuum at room temperature. No difference is observed on the corresponding images for before and after electron beam irradiation, indicating the electron beam damage effect is negligible.

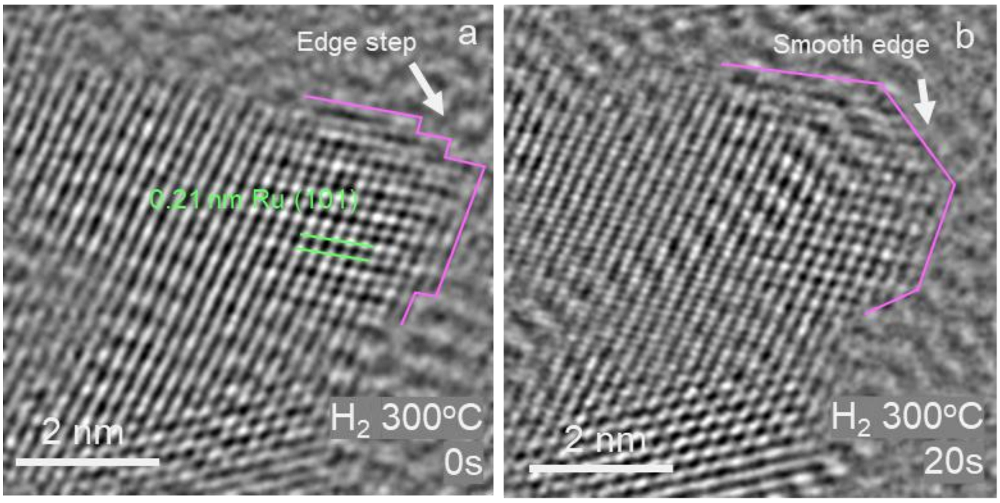


Figure S4 The *in situ* HRTEM image of Ru/$Al_2O_3$ in $H_2$ atmosphere at 300 ºC for 0s (a) and 20s (b)

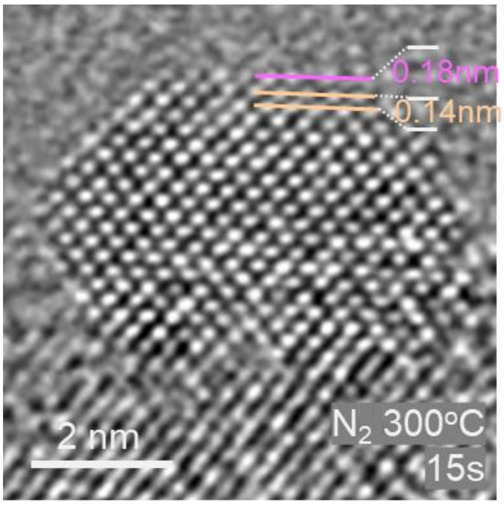


Figure S5 The *in situ* HRTEM image of Ru/$Al_2O_3$ in $N_2$ atmosphere at 300 ºC for 15s

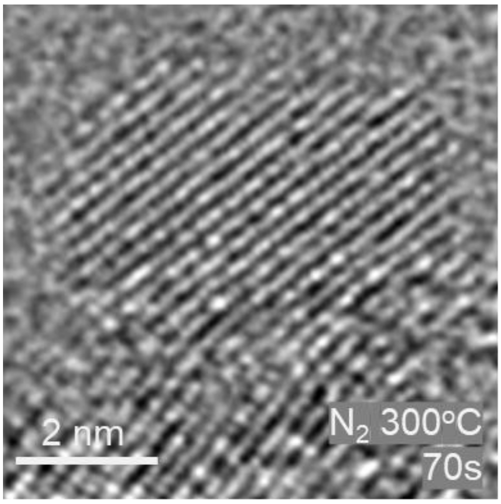


Figure S6 The *in situ* HRTEM image of Ru/$Al_2O_3$ in $N_2$ atmosphere at 300 ºC for 70s

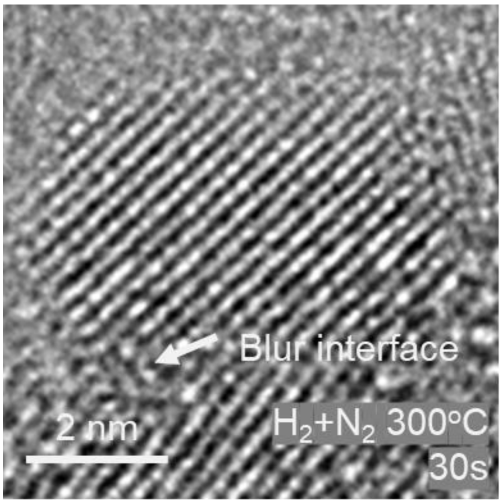


Figure S7 The *in situ* HRTEM image of Ru/$Al_2O_3$ in $H_2$ and $N_2$ atmosphere at 300 °C for 30 s.

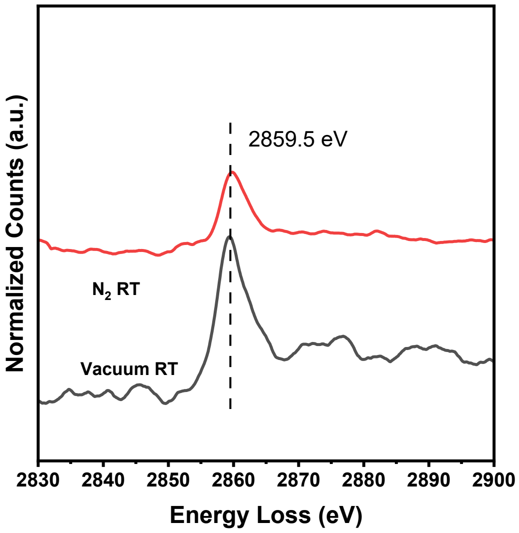


Figure S8 Ru-L EELS spectra of Ru/$Al_2O_3$ in vacuum or in $N_2$ atmosphere at room temperature.

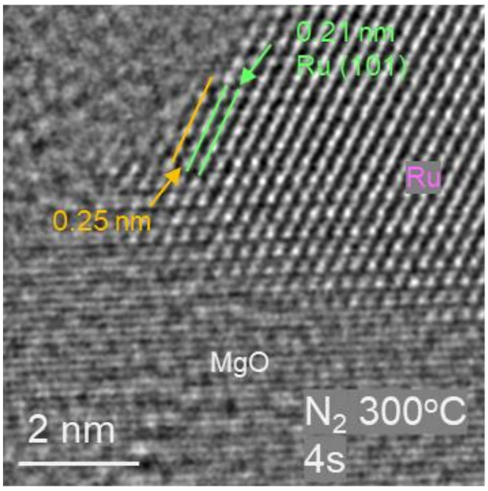


Figure S9 *in situ* HRTEM image of Ru/MgO heated in $N_2$ at 300 °C

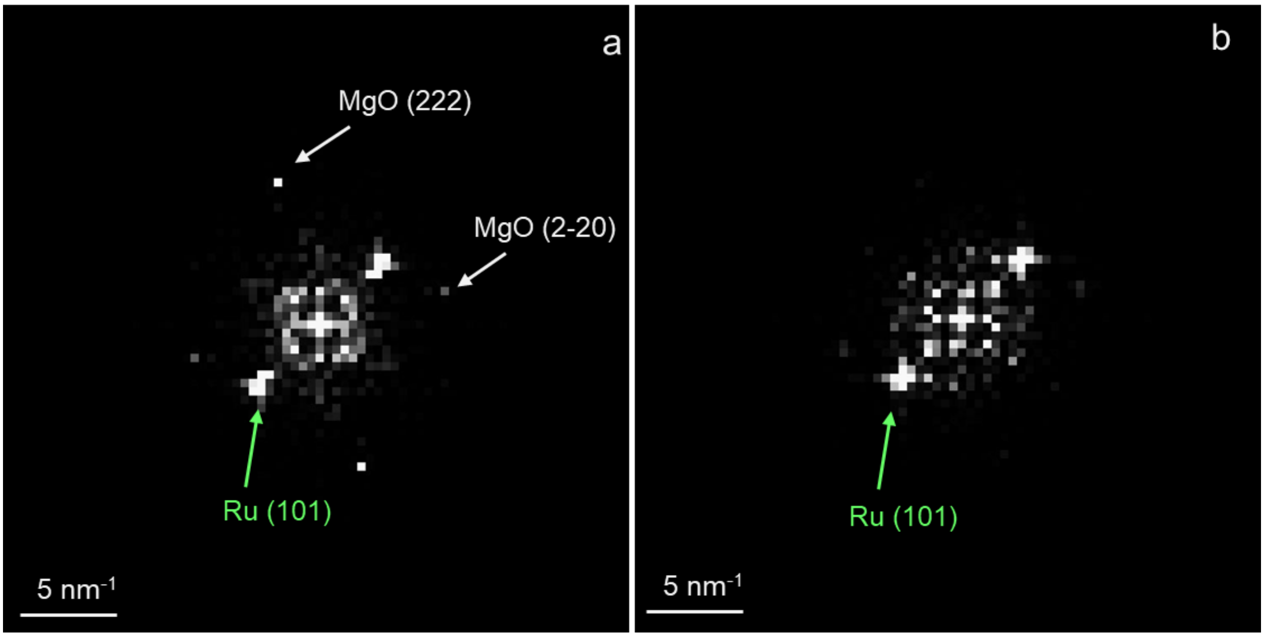


Figure S10 The FFT of *in situ* ETEM images acquired on the edge of Ru/MgO at 300 ºC at 0s(a) and 120s (b) in $N_2$ and $H_2$ atmosphere.

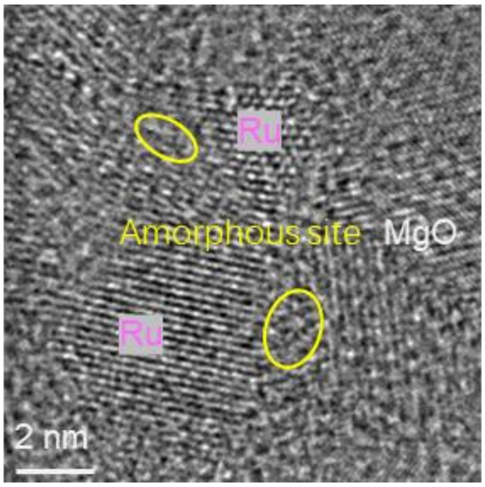


Figure S11 *in situ* ETEM image of Ru/MgO after being treated in $H_2$.

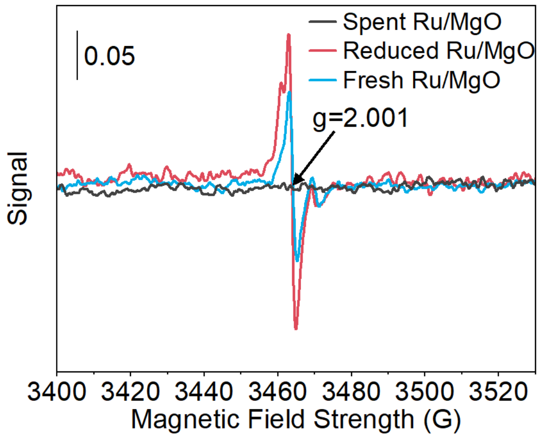


Figure S12 EPR spectra of fresh Ru/MgO, reduced Ru/MgO, and spent Ru/MgO catalysts.

The EPR results of fresh and spent Ru/MgO determined a peak located at g=2.001, which is corresponding to the oxygen vacancies on the surface of MgO.[10] Compared to the fresh sample, the reduced sample displayed a larger peak, suggesting a higher concentration of oxygen vacancies. This provides solid evidence that reduction treatment will introduce a surface oxygen vacancy on the surface of MgO. It is also notice that spent Ru/MgO doesn't exhibit the g=2.001 peak. This may originate from the insertion of N* into surface oxygen vacancies on MgO and refill the oxygen vacancies.

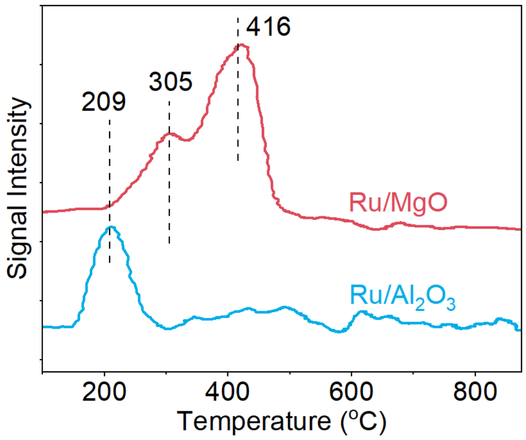


Figure S13 $H_2$-TPR profile of Ru/MgO and Ru/$Al_2O_3$

The $H_2$-TPR result for Ru/MgO exhibits the peaks located at 305 °C and 416 °C, which are attributed to the formation of $Ru^0$ and the hydrogen spillover, respectively.[11,12] In contrast, the $H_2$-TPR result of Ru/$Al_2O_3$ only shows a single reduction peak at 209 °C, which is corresponding to the Ruthenium reduction.[13]

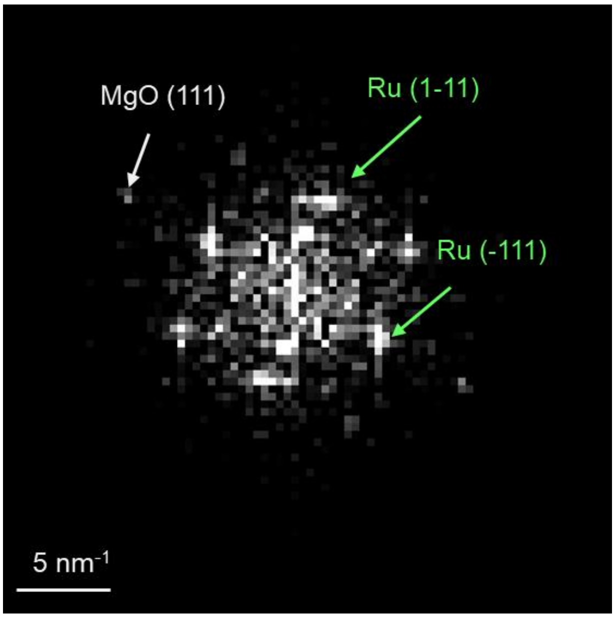


Figure S14 *in situ* ETEM image of Ru/MgO at 400 ºC for 50s in $N_2$ and $H_2$ atmosphere.

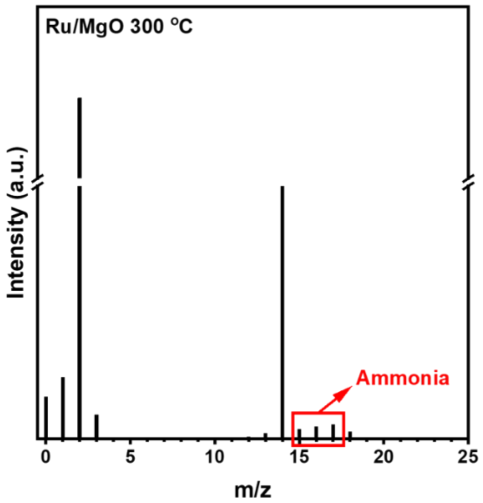


Figure S15 the MS spectra of product gas from ETEM at 300 ºC when both $N_2$ and $H_2$ were introduced to Ru/MgO.

We noticed a relatively strong signal at m/z =17 from the signal from MS spectrum. Although the m/z=17 intensity maybe influenced by the existence of water, in this spectrum, the m/z=17 signal contributed to water is limited as the signal at m/z=18 is low. Theoretically, the m/z=17 signal from water should only be 20% of signal intensity at m/z=18. Thus, this spectrum gives solid evidence that $NH_3$ was produced during the ETEM test.

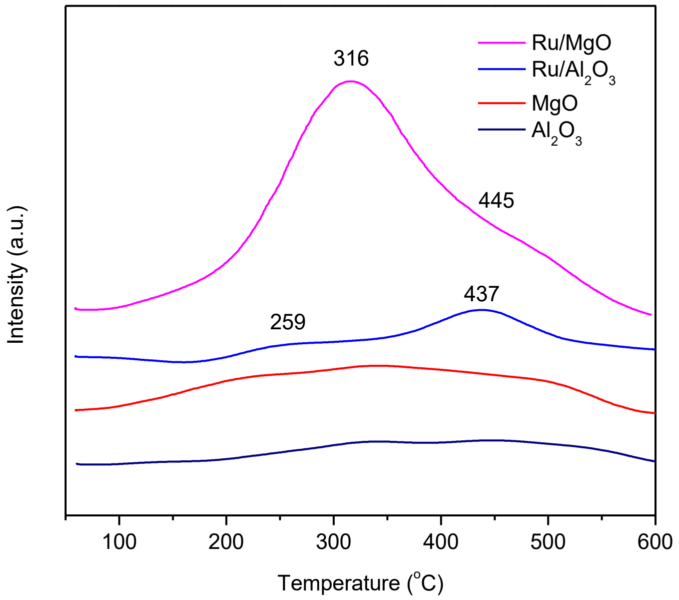


Figure S16 The $N_2$-TPD for Ru/MgO. Ru/$Al_2O_3$, MgO and $Al_2O_3$

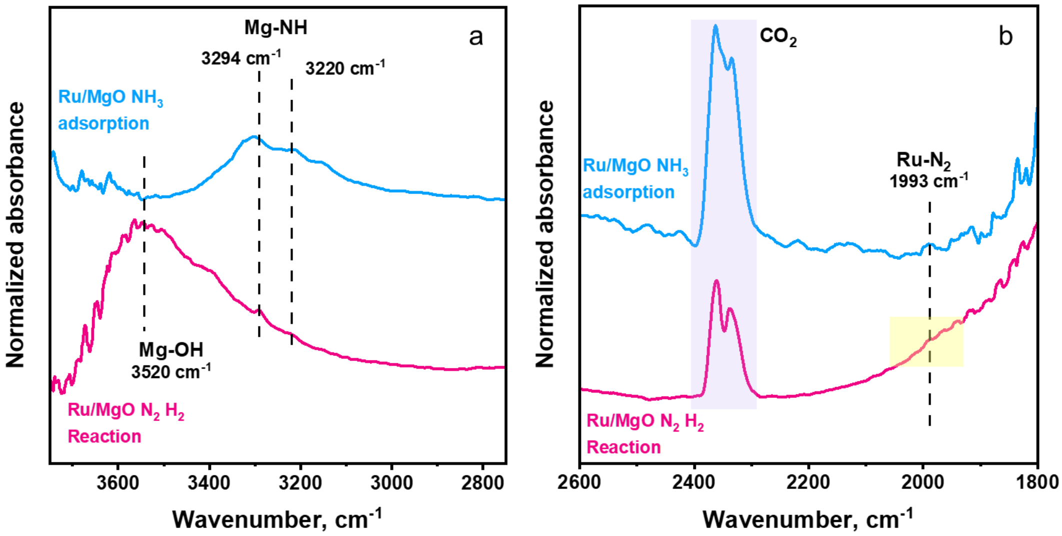


Figure S17 *in situ* DRIFTS spectrum in the range between 3750~2750 cm $^{-1}$ (a) and 2600~1800 cm$^{-1}$ of Ru/MgO for $NH_3$ adsorption (blue) and the *in situ* DRIFTS spectrum of Ru/MgO reacted in $N_2$ and $H_2$ environment at 300 °C (magnet).

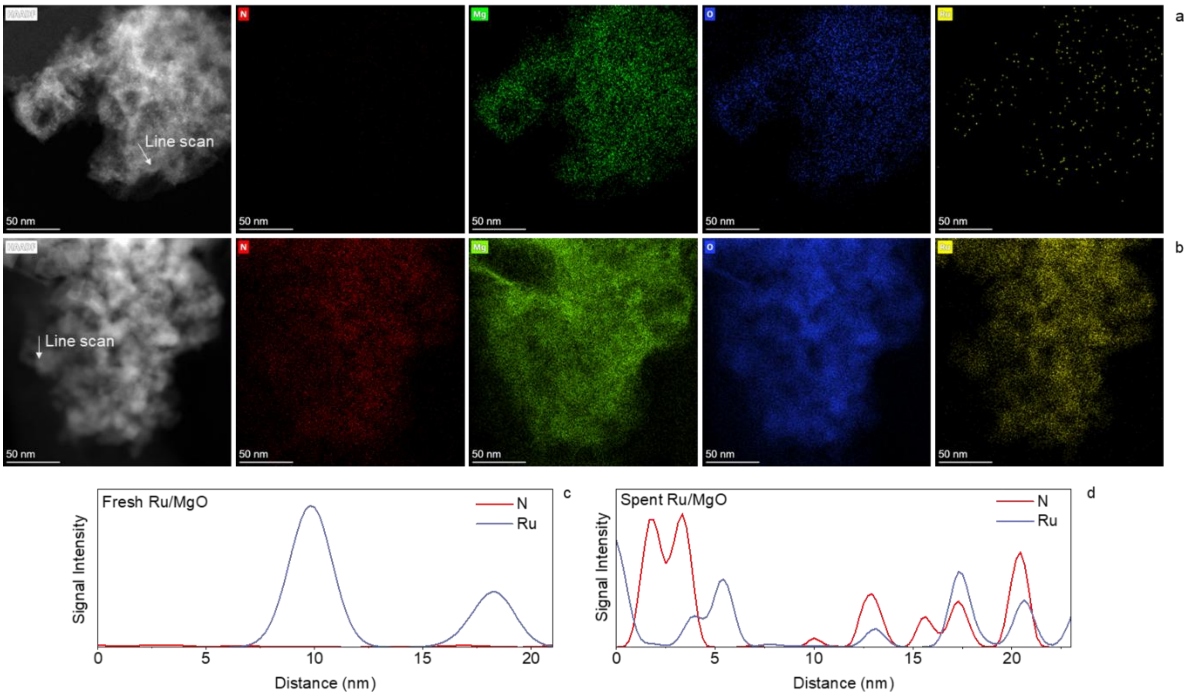


Figure S18 STEM-EDS mapping of fresh Ru/MgO (a) and spent Ru/MgO (b). The line scan results of N and Ru signal for Fresh Ru/MgO (c) and spent Ru/MgO (d)

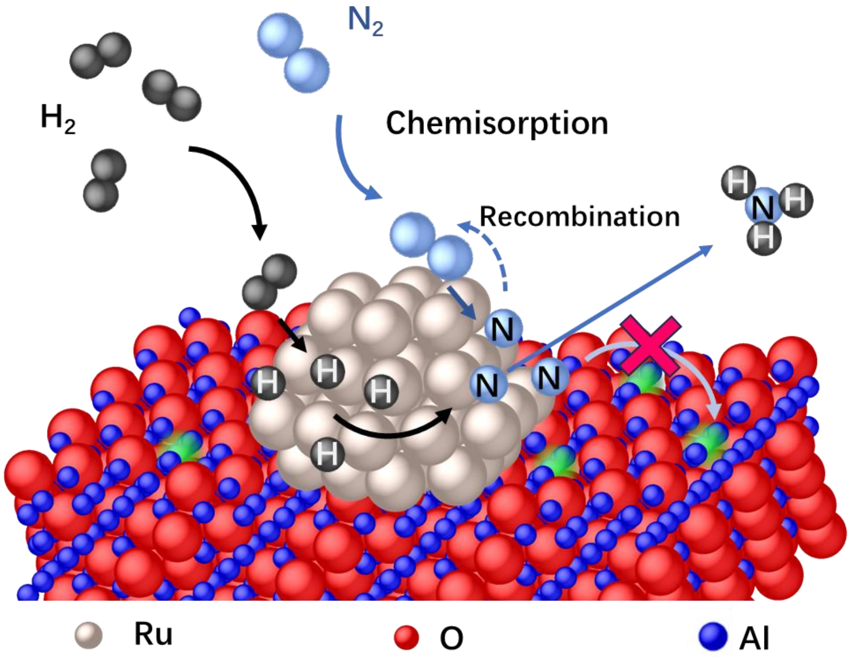


Scheme S1 the reaction pathway over Ru/$Al_2O_3$ which activated $N^*$ migration is limited.

# Reference:


1 He, D. *et al.* Promoter effects on nickel-supported magnesium oxide catalysts for the carbon dioxide reforming of methane. *Energy & Fuels* **31**, 2353-2359 (2017). https://doi.org:10.1021/acs.energyfuels.6b02361

2 De Backer, A., van den Bos, K. H. W., Van den Broek, W., Sijbers, J. & Van Aert, S. StatSTEM: An efficient approach for accurate and precise model-based quantification of atomic resolution electron microscopy images. *Ultramicroscopy* **171**, 104-116 (2016). https://doi.org:https://doi.org/10.1016/j.ultramic.2016.08.018

3 Kohn, W. & Sham, L. J. Self-consistent equations including exchange and correlation effects. *Phys Rev* **140**, A1133-A1138 (1965). https://doi.org:10.1103/PhysRev.140.A1133

4 Kresse, G. & Furthmüller, J. Efficiency of ab-initio total energy calculations for metals and semiconductors using a plane-wave basis set. *Comput Mater Sci* **6**, 15-50 (1996). https://doi.org:10.1016/0927-0256(96)00008-0

5 Grimme, S., Antony, J., Ehrlich, S. & Krieg, H. A consistent and accurate ab initio parametrization of density functional dispersion correction (DFT-D) for the 94 elements H-Pu. *J Chem Phys* **132** (2010). https://doi.org:10.1063/1.3382344

6 Perdew, J. P., Burke, K. & Ernzerhof, M. Generalized gradient approximation made simple. *Phys Rev Lett* **77**, 3865 (1996). https://doi.org:10.1103/PhysRevLett.77.3865

7 Kresse, G. & Joubert, D. From ultrasoft pseudopotentials to the projector augmented-wave method. *Phys Rev B* **59**, 1758 (1999). https://doi.org:10.1103/PhysRevB.59.1758

8 Pauling, L. Atomic radii and interatomic distances in metals. *J. Am. Chem. Soc.* **69**, 542-553 (1947).

9 Bergeret, G. & Gallezot, P. Particle size and dispersion measurements. *Handbook of heretogeneous catalysis* **2**, 738-765 (2008).

10 Giamello, E., Paganini, M. C., Murphy, D. M., Ferrari, A. M. & Pacchioni, G. A Combined EPR and Quantum Chemical Approach to the Structure of Surface Fs+(H) Centers on MgO. *J. Phys. Chem. B* **101**, 971-982 (1997). https://doi.org:10.1021/jp962619m

11 Mori, K., Miyawaki, K. & Yamashita, H. Ru and Ru–Ni Nanoparticles on $TiO_2$ Support as Extremely Active Catalysts for Hydrogen Production from Ammonia–Borane. *ACS Catal.* **6**, 3128-3135 (2016). https://doi.org:10.1021/acscatal.6b00715

12 Ju, X. *et al.* Highly Efficient Ru/MgO Catalyst with Surface-Enriched Basic Sites for Production of Hydrogen from Ammonia Decomposition. *ChemCatChem* **11**, 4161-4170 (2019). https://doi.org:https://doi.org/10.1002/cctc.201900306

13 Jiménez-Barrera, E. *et al.* CO/$H_2$ adsorption on a Ru/$Al_2O_3$ model catalyst for Fischer Trospch: Effect of water concentration on the surface species. *Appl. Catal., B: Environ.* **237**, 986-995 (2018). https://doi.org:https://doi.org/10.1016/j.apcatb.2018.06.053
